# Institutional Place Identity and Life Adaptation among Elderly People in Taiwan

**DOI:** 10.3390/geriatrics7020039

**Published:** 2022-03-29

**Authors:** Ning-Chun Chuang, Pei-Chun Kuo, Yi-Wen Chiu

**Affiliations:** 1 Center of Rare Disorder, National Taiwan University Hospital Yunlin Branch, Yunlin County 64041, Taiwan; maynin2001@gmail.com; 2 Department of Nursing, National Taiwan University Hospital Yunlin Branch, Yunlin County 64041, Taiwan; candy73723@gmail.com; 3 Department of Nursing, Chung Shan Medical University & Chung Shan Medical University Hospital, Taichung 40201, Taiwan

**Keywords:** place identity, life adaptation, long-term care institutions

## Abstract

Background: Many elderly people in Taiwan move to institutional care due to disability or insufficient family resources. This study aimed to understand the place identity and life adaptation of institutional residents and their influencing factors, and to explore the correlation between these two. Methods: This study adopted a cross-sectional survey method. A total of 120 cases were collected with structured questionnaires, and SPSS 22.0 software package was used for statistical analysis. Results: The place identity was the highest in the sense of belonging, while the sense of participation was the worst; adaptation to life was the best in terms of care management, and the worst in terms of adaptation to life and assistive devices. The length of stay in the institution, daily activities, and the number of chronic diseases were significantly different from place identity, and the number of chronic diseases was significantly different from life adaptation. Conclusions: The results of this study can be used as a reference for institutions to improve the quality of care. It is suggested that institutions can organize more activities to promote interaction and participation among residents, to strengthen their local identity and life adaptation.

## 1. Introduction

With the advancement of medical technology, the average life expectancy has increased and the disease patterns of the elderly population have gradually become chronic. Countries around the world have entered an aging society, and the elderly population has even grown in multiples. The United Nations estimates that the global elderly population will reach 850 million in 2025 and 1.55 billion in 2050 [1]. Taiwan’s elderly population is not far behind, and older people over 65 represented 16.07% of the total population in 2020 [2], the aging index reached 127.8%, and it is estimated that by 2026, Taiwan’s elderly population will exceed 20%, enter a society of super-aged, and the aging index will reach 441.8% in 2060 [3]. The Aging Index refers to the number of elderly population (65 years and older) per 100 individuals younger than 14 years of age in a specific population. Thus, the higher the index is, the older the population results. Therefore, the rising aging index reflects the increasingly obvious situation of Taiwan’s “senior ageing and fewer children”. With the continuing low fertility rates, the average working population is subjected to the economic pressure of the upper and lower generations of dependent populations. It will also be more serious, which means that the working population will gradually decrease in the future, and it will not even be able to meet the needs of the elderly population over the age of 65. When compared to Western countries, Taiwan is obviously late in entering an aged society, but whether it is in terms of the “quantity” or “structure” of the population, Taiwan is facing a very rapid population change. Various care issues derived from the aging society have become the focus of global attention, especially the living arrangements of the elderly, which is an important issue that requires the most thought and planning.

The concept of filial piety is a stable value in Chinese culture and is also an important factor that affects intergenerational living arrangements [4,5]. Parent–child cohabitation is one of the unique norms of oriental culture, and the living arrangements of the elderly in Taiwan have long been inconsistent. There has been little change, and most people expect to live with their children [6,7], and the elderly who live in institutions only account for 1.5% of all elderly people [8]. However, the average household size in Taiwan has been decreasing year by year. The average household size in 2020 is only 2.67 [9]. Therefore, care and living arrangements for the elderly need to be considered. In the past, Taiwanese society did not pay much attention to institutional care. The general public or the elderly often viewed staying in an institution as a sign of poverty, illness, or abandonment. Residents were usually unable to take care of themselves due to illness or daily life. However, the number of long-term care institutions in Taiwan continues to grow [10]. It can be seen that although institutional care is not the mode of living arrangement for most elderly people, it is indispensable; especially when the family is not sufficient to cope with or bear the heavy responsibility of care, institutional placement may be the best option.

Migrating and adapting to a new environment is a rather difficult process for the elderly [11,12], and poor adaptation can affect their health and quality of life [12]. In the past, Taiwan’s studies on the adaptation of the elderly to institutions have shown that the elderly adapt mainly by ‘resigning their fate’, which is full of helplessness and does not entail having a sense of belonging to the institution [13,14]. The most important factor that influences the elderly in the process of adapting to institutional life is cultural values and social background [15,16], which are the glue and basis for identifying or connecting with a place. Although the causes and mechanisms of the connection between places are still unclear, research [17] has pointed out that the connection between places, or between people and places, has a positive impact on people. If people have a strong psychological connection to the living environment, the living environment will be full of positive experiences.

The occurrence of “place identity” must be a psychological process of incorporating oneself into the place, experiencing the environment and activities, and perceiving oneself to belong to this place, and then transforming it into memory through emotion, and generating a sense of belonging to this special place [18]. Entriken [19] also believes that the “place identity” is similar to a group of people, events, and activities. When people start to contact a special place, they will have an emotional connection to the place. When the frequency of contact increases and they are satisfied with the services, they will then generate belonging. Only when it is identified with trust and willing to pay and maintain for the place can it be called “place identification” [20]. Therefore, it can be seen that “place identity” takes a period of time to experience, and it can only be generated after emotional belonging to the place takes places. The personal psychological process of belonging and identification, and then having a positive attitude, results in being willing to give practical actions to participate. Based on the above literature, it can be seen that place identity basically includes three aspects: “sense of belonging”, “sense of caring”, and “sense of participation”.

Additionally, “life adaptation” refers to the interactive relationship between the individual and the living environment. This interactive relationship is in a good ideal state for the individual, the individual and the environment, the relationship between themselves, others, and the group, as well as the psychological and emotional behavior. It is also a process of individual adjustment in the environment to enhance personal ability, satisfaction, and self-realization, and to achieve a harmonious relationship between the individual and the environment [21,22]. For the elderly, adaptation to life is a very subjective cognition, including physical health, inner self-feeling, personal and external social interaction, and is achieved through the interaction between individuals and life, the environment, interpersonal relationships, and medical care [23]. As Chen et al. [24] clearly pointed out, life adaptation should include adaptation to environmental structure, interpersonal and social relationships, life and accessories, and long-term care management.

The aging of the global population is progressing rapidly, and the greatest impact of the increasingly large elderly population on the country and society is the increase in the burden of medical care and social care. Taiwan has entered an aged society, and the family structure and concept of filial piety have changed. After the elderly are disabled, they are faced with a shortage of family caregivers. Family members turn to institutions to replace most of their caregiving responsibilities. For elderly living in institutions, it is an important turning point in life. Familiar life isolation forces one to reface and adapt to a completely unfamiliar environment. In the past, few people have seriously understood, in the process of adapting to the elderly, what mechanism is used to allow them to slowly adapt to the new environment and take into account their psychological feelings and their views on aging. Therefore, this study started from the perspective of the place identity of the elderly to explore and understand their adaptation situation. It is hoped that through the research results, the service quality of institutions can be integrated and promoted, and the power of connection between the body, mind, community, and environment of the elderly can be provided, so that the institution can become the last “home” for the elderly to live in.

Currently, there are few studies on the correlation between place identity and adaptation to elderly life in institutions. Therefore, the authors wanted to further explore this topic.

In summary, the purpose of this study was to:

(1) Understand the status of local identity and adaptation of life of the residents of the institution.
(2) Explore the relevant factors that affect the local identity and adaptation to life of the institution residents.
(3) Explore the correlation between institutional residents’ place identity and life adaptation.

## 2. Materials and Methods

In line with the above research purposes, the research questions raised in this study are as follows:

(1) What was the identification status of the residents with a sense of belonging, caring, and participation in organizations?
(2) What was the adaptation of the residents to the environmental structure, life and accessories, care management, and interpersonal social relationship of institutions?
(3) What were the relevant demographic characteristics and health conditions that influence the place identity of the residents?
(4) What were the relevant demographic characteristics and health conditions that influence the life adaptation of the residents?
(5) What was the correlation between institutional residents’ place identity and life adaptation?

In order to answer the above research questions, this study adopted a cross-sectional survey method to conduct a convenient sample of residents who had been living in institutions for more than a month in the Yunlin area of Taiwan Province. Residents over 65 years old, with clear consciousness and no language or text communication barriers were selected as subjects. A total of 120 inhabitants were actually collected. Data collection was conducted with the same researcher and face-to-face interviews. Since it is difficult to have general social interaction among strangers, in order to allow the interviewees to fully trust the researcher, express their true feelings, and answer questions, the researcher entered the field as an intern student. The researcher and the interviewee established a familiar, respectful, and equal communication relationship before collecting data. During the actual interview, it was explained to the interviewee that their personal information was to be encoded in an anonymous way to protect their privacy, and their right to care will never be compromised. At the same time, the researchers also ensured and controlled the interview situation without the presence of a third person, to reduce the influence of socially desirable responses.

The measurement tools of the research were divided into three parts: personal basic information, place identity, and life adaptation. In other words, this study used two main scales: ‘place identity subscale’ and ‘life adaptation subscale’. The place identity subscale is a self-developed structural scale based on the references, including three dimensions of a sense of belonging, a sense of caring, and a sense of participation, with a total of 15 questions. The Likert scale five-point scoring method was adopted. A higher score indicated the higher identity of the residents with the institution. Another research scale, the life adaptation subscale (total score: 25–100), adopted the scale developed by Chen in 2015 [24], and was used with her authorization. This scale includes four aspects of environmental structure, life and assistive services, care management, and interpersonal and social relationships. The total number of questions is 25, and the scoring method was also based on the Likert scale. The higher the score, the better the residents adapt to the life of the institution.

The scale was verified by experts for content validity after completion. This study invited 5 experts in the relevant long-term care field to give professional opinions on the suitability of the content of this questionnaire and the clarity of the text. The scoring method for each question was calculated on a scale of 1–4 to evaluate the effectiveness of the tool. The higher the score, the better the applicability of the subject. This research first calculated the Content Validity Index (CVI) of each question. If the number of experts with 3~4 points/total number of experts ≥0.8, the question was reserved. Finally, the CVI of the overall scale was calculated to establish the content validity index. The CVI of the overall scale was 1, the CVI of the place identity subscale was 0.95, and the CVI of the life adjustment subscale was 0.94. In terms of internal consistency reliability, Cronbach’s α value of the overall scale was 0.89 and the Cronbach’s α value of the local identity subscale was 0.88–0.95. Cronbach’s α values of the life adjustment subscale ranged from 0.70 to 0.93 (Cronbach’s alpha of the original scale is 0.66~0.95), indicating that this scale had good reliability and validity.

This study was carried out after the review and approval of the Research Ethics Review Committee of the Yunlin National Taiwan University Hospital (No. 202010060RIND). After data collection was complete, the SPSS 22.0 statistical software was used for coding and documentation, and descriptive statistics and inferential statistics were performed according to the research objectives and variable characteristics.

## 3. Results

There were 58 male (48.3%) and 62 female (51.7%) subjects in the study, with an average age of 78.8 ± 9.1 years old. 58.3% were widowed, 85.8% had an education degree below primary school, 82.5%, with ordinary economic status, accounted for the most, and 114 people (95.5%) had religious beliefs. The average time for residents to stay in institutions was 31.8 months (about 2 years and 8 months). An amount of 85% (102 people) of the residents have stayed in institutions for less than 5 years; especially, those who stayed for 1–5 years accounted for more than 55.0%, and for 85.8% of them it was the first time they had stayed in an institution, without previous experience in other institutions (Table 1).

In terms of health status, the average ADL score was 62.9 (SD = 24.1) and 103 patients (87.5%) were moderately and severely dependent. The mean IADL score was 5.7 (SD = 4.9). The average number of patients diagnosed with chronic diseases was 1.84 (SD = 1.0) and 56 patients were diagnosed with two diseases (Table 1).

### 3.1. Score of Place Identity and Life Adaptation

The average score on the place identity scale was 47.7 and the standard deviation was 9.30, indicating that the overall place identity of the subjects in the institution was at a medium to high level. From the perspective of the three dimensions of “sense of belonging”, “sense of caring”, and “sense of participation”, the scores in order were “sense of belonging”, “sense of caring”, and “sense of participation” (Table 2), indicating that the “sense of belonging” felt by residents of institutions was superior to “sense of caring” and “sense of participation”.

The total mean score and standard deviation of the life adaptation scale were 79.6 and 9.22, indicating that the life adaptation of all subjects was in the medium to high range degree. From the four dimensions of “environmental structure”, “life and accessories”, “care management”, and “interpersonal and social relations”, the scores were in order of “care management”, “interpersonal and social relations”, “environmental structure”, and “life and accessories”, indicating that the subjects had the best adaptability in “care management”. This is better than “interpersonal social relationship”, “environmental structure”, and “life and accessories” (Table 2).

This section may be divided by subheadings. It should provide a concise and precise description of the experimental results, their interpretation, as well as the experimental conclusions that can be drawn.

### 3.2. Inferential Statistics Analysis

#### 3.2.1. Differences between Personal Characters and Place Identity/Life Adaptation

Further analysis of the differences between the personal characters of the residents and the total score of the identity of the place showed that the time staying in the institution, the status of daily activities (ADL), and the number of chronic diseases were significantly different from the total score of place identity. Those who had been in the institution for more than 5 years had a significantly higher place identity score than those who had stayed in the institution for less than 1 year; those who were moderately dependent on daily activities had significantly higher total place identity scores than those who were heavily dependent (≤60 points) (Table 3).

There were significant differences in institutional stay time, ADL, and the number of chronic diseases, as well as the place scores. According to Scheffe’s post hoc comparative analysis, the score of care and participation for institutions in those who stayed in institutions for longer than 5 years was significantly higher than that in those who stayed in institutions for less than 1 year. Those with moderate dependence (ADL 61–90 points) had higher care and participation scores than those with severe dependence (ADLs 60 points), and those with mild dependence (ADLs 91 points) had higher participation scores than those with severe dependence. The care scores in subjects suffering from one chronic disease were significantly higher than those suffering from two chronic diseases, and the participation scores in subjects suffering from no chronic disease were significantly higher than those suffering from two chronic diseases and more than three chronic diseases (Table 3).

There was a significant difference between the number of chronic diseases and the total adjustment score of life among the residents. The total adjustment score of residents with a chronic disease was significantly higher than that of subjects suffering from two chronic diseases. According to the four dimensions of the life adaptation subscale, there were significant differences in the age and life adaptation subdimension of the residents, and Scheffe’s post hoc comparative analysis showed that the scores of the life and accessories of 65–74 years were significantly higher than those of 75–84 years old. However, there were no significant differences in environmental structure score, care management score, or interpersonal and social relationship score (Table 4).

There were significant differences between ADL and the life adjustment scale. The better the ADLs, the better the adaptation of the environmental structure. There were significant differences in the scores for the three dimensions of environment structure, life and accessories, and care management in the number of chronic diseases. The lower the number of chronic diseases, the better the adaptation to environmental structure, life and accessories, and care management (Table 4).

#### 3.2.2. Correlation Analysis between Place Identity and Life Adaptation

Pearson’s correlation of product differences was used to analyze the correlation between place identity and life adaptation. Table 5 showed that the total score of life adaptation was not correlated with the sense of participation in the identity of the place, and there was no correlation between “care management”, “interpersonal and social relationships”, the total score of the identity of the place, and its three subdimensions of “sense of belonging”, “sense of caring”, and “sense of participation” of life adaptation. However, the “environment structure” of life adaptation was positively correlated with the “sense of belonging” (*R* = 0.28, *p* < 0.01), “sense of caring” (*R* = 0.27, *p* < 0.01), “sense of participation” (*R* = 0.33, *p* < 0.01), and the “total score of place identity” (*R* = 0.36, *p* < 0.001). “Life and accessories” were positively correlated with “sense of belonging” (*R* = 0.36, *p* < 0.001), “sense of caring” (*R* = 0.34, *p* < 0.001), “sense of participation’ (*R* = 0.29, *p* < 0.01), and “total score of identity of the place’ (*R* = 0.39, *p* < 0.001). The better the place identity of “belonging”, “caring”, and “participation”, the better the “environment structure” and “life and accessories”. Meanwhile, the total score of ‘adaptation to life” was positively correlated with ‘sense of belonging” (*R* = 0.30, *p* < 0.01), “sense of caring’ (*R* = 0.26, *p* < 0.01), and “total score of identity of place” (*r* = 0.26, *p* < 0.01)

## 4. Discussion

In terms of place identity score, the “sense of belonging” aspect of this study has the highest score. Hagerty et al. [25] pointed out that there are two attributions of belonging, one is a feeling of being valued, needed, and respected by others or organizations, and the other is a harmonious coherence with others or organizations through shared or complementary processes. When residents feel the support, respect, and acceptance of the organization, a sense of satisfaction and a sense of psychological coherence will be triggered psychologically. Because the residents of this study were cared for 24 h in their daily lives, they will also feel positive support and be influenced psychologically and ideologically, and gradually develop their identity with the institution [26]. The sense of belonging is also in a state of continuous and cumulative influence. The key to appearing and being perceived is often the context behind the existence of people [27]. In this study, residents stayed in institutions for nearly three years on average. Residents may have gradually accepted that the institution is the final place for them to settle down. Furthermore, the residents had high homogeneity. They were all from similar backgrounds who needed help from others to live, had no family companionship, and their daily lives were closely integrated with a common normative. High cohesion will also make members feel a strong sense of belonging [28]. Other studies have pointed out [29,30] that if residents have exclusive space or seats in the institution and have other free time to allow residents to do what they want, with autonomy and a sense of achievement, the elderly will create a sense of belonging similar to that of “home”. Residents of the institutions in this study have their own rooms, although not all of them are single rooms, but still have their own independent space or fixed seats, and the staff of the institution can also call each of them Residents know each resident, which may be the reason why residents score higher on the “belonging” dimension than other dimensions.

This study shows that the longer the stay, the fewer the number of chronic diseases and the better the function of daily activities, then older people have a better overall sense of identity with the institution. Echoing the statement of McShane et al. [28], the longer people live in a certain place, the higher their identification with the place. Meanwhile, this may be because the longer the stay, the more the residents know each other, and the more they interact and share [20]. Those with fewer chronic diseases or better daily activities represent a better health status. Therefore, they have more positive feedback on their external behaviors and inner feelings and are more likely to pay attention to the big and small matters of the organization [26]. So, the overall sense of identity of the organization is better. However, there was no difference between the age and place identity of the residents in this study, which was different from the study of Pretty et al. [31]. Their research showed that older residents have a greater degree of place identity than the younger residents. The possible reason was that the subjects in this study were all over 65 years old. For the elderly here, although there were young elderly and old elderly, the age gap was not as large as the research object of Pretty et al. [31], and basically still belonged to the same generation.

In terms of adaptation to life, the highest score in this study is “care management”. This may be because the pace of life in the institution is quite regular and structured, and the daily activities are almost all in accordance with the schedule. These deterministic services and life can make institution residents feel at ease and gain spiritual stability [32]. Moreover, care management is the most important part of the life of residents, and residents must adapt themselves in the shortest possible time. The second highest score is “interpersonal and social relations”, probably because long-term care institutions are mainly group living, and residents in the institutions get along with each other 24 h a day. When people accompany each other, they naturally interact and chat, and it is easier to care for and support each other [20]. On the other hand, “environmental structure” and “life and accessories” were ranked third and fourth, probably because 30% of the residents of this study stayed for less than a year, so they may have a low degree of familiarity with the institution or recognition of the living environment. Additionally, because institutions cannot provide personal aids or the aids provided (e.g., wheelchairs) are insufficient and inappropriate, this may also be a factor leading to its lower score. Furthermore, institutions are not like their own families; the furnishings and decoration of the environment could not be easily changed, nor could they arrange or place personal furniture and favorite items according to their own preferences, and the relative adaptability would therefore be poor [33].

The age, gender, marital status, etc. of the residents in this study are not statistically different from the adaptation to life, which is consistent with the results of many studies [13,14,16]. However, this is different from the study by Lin et al. [34], who found that the higher the level of education, the better the adaptation of the residents. The possible reason for the difference is that the elderly in Taiwan have more negative views or see a last resort option in the institutions. The higher the education level, the more oppressive they feel in institutional life. There are significant differences in the number of chronic diseases, the functions of daily activities, and the adaptation of life. It may be because the more chronic diseases, and the more common uncomfortable situations or symptoms are, the worse life satisfaction and life adaptation will be. The better the health of the residents, the greater their ability, so their adaptation to life will be better [14].

Whether the elderly were there due to physical limitations, insufficient family care resources, or active or passive admission to institutions, for them, accepting placement and care means that they must gave up all the familiar living patterns. The unfamiliar environment and staff of the institution were a shock to the residents. The research of Tsai et al. [35] pointed out that if the elderly can establish a close emotional connection and an attachment relationship with familiar people and establish a support system, they will meet the psychological needs of the elderly and improve their life satisfaction. If there are no familiar members in the environment and there is no way to get along with acquaintances, the loneliness of the residents will increase [36]. In this study, residents lived in institutions for an average of nearly three years, but the overall place identity and life adaptation scores were only at a moderate level, and there were still many things that could be improved. In the process of this research, it was found that although the staff could correctly address each resident by name and understand each resident’s habits, hobbies, or favorite locations, the residents did not have the same cognition. Residents only knew who the director of the institution is, and can also distinguish who is nursing staff and who is a nursing attendant from the uniforms they wear (for example, those who wear bibs are nursing attendants). Most of the staff members are collectively referred to as “Miss” or “Girl”, and the residents cannot clearly identify or call each one. During interviews, the researchers also heard the residents express that the caregivers are different every day, and that no matter who takes care of them, it is the same. This may also be the reason why place identity and adaptation to life are moderate.

## 5. Conclusions and Suggestions

This study found that the number of elderly women and men living in institutions was similar (62 vs. 58), with an average age of 78.8 years. Most of them were widows, those with little to no primary school education, and those with moderate to severe dependence. Residents stayed in the institution for an average of 31 months (approximately 2 years and 7 months), and the average number of chronic diseases was 1.84. In line with the three objectives of this study, the conclusions of this study are as follows:

1. The local identity and life adaptation of the residents are at the upper-middle level (the percentage of scores are 63.6 and 79.6% respectively). The average scores of the three dimensions of place identity are “sense of belonging”, “sense of caring”, and “sense of participation”, that is, the residents of the institution have the highest sense of belonging to the organization, and the least sense of participation in the organization. As for the four dimensions of life adaptation, residents have the best adaptation to the institution’s “care management”, followed by “interpersonal social relations” and “environmental structure”, and the worst adaptation is the “life and accessories” dimension.
2. In terms of basic personal information, the time of staying in the institution (*F* = 3.79, *p* < 0.05), the number of chronic diseases (*F* = 4.33, *p* < 0.01), and the status of daily activities (*F* = 8.67, *p* < 0.001) is significantly different from “place identity”. The longer residents stay in the institution, the better the “place identity”, and those with fewer chronic diseases and better functions of daily activities have better “place identity”. In terms of life adaptation, only the number of chronic diseases and the “life adaptation” of institutional residents had significant differences (*F* = 5.35, *p* < 0.01), and the more chronic disease diagnoses, the worse the “life adaptation”.
3. The “total life adaptation score” has a significant positive correlation with the “belonging” and “caring” aspects of place identity and the “overall place identity” score. That is to say, the better the residents’ “sense of belonging”, “sense of caring”, and “overall place identity” to the institution, the better their “overall life adaptation”.

Based on the above conclusions, this study suggests that for the elderly who suffer from various chronic diseases and have poor daily activities, the institution’s staff should provide more care and assistance to improve their physical and mental security, and establish a special division of labor when residents move in. Since most of the staff in the organization are in a shift system, for the residents, the care staff may be different every day, and it takes a long time to become familiar with the environment and establish a relationship between the staff. There may also be repeated questions and answers between the staff and the residents. The phenomenon is relatively unfavorable to the adaptation and identification of residents. If the concept and atmosphere of “My Nurse, My Caregiver, My Resident” can be created, so that each resident has their own dedicated staff, creating a sense of intimacy and trust, this may be favorable. In this way, the relationship between residents and staff is one-to-one, and comprehensive responsibility, continuity, coordination, autonomy, and individualized care may be more effective in promoting residents’ identification. Furthermore, residents can also get to know the staff of the institution as soon as possible, reducing the multiple confusions of residents who need to adapt to the institutional environment and personnel at the same time. In terms of planning of life, it is also suggested that institutions organize diversified activities as much as possible, and create more topics similar to the experiences of the past of the residents in the activities to stimulate the daily life of the elderly and promote their participation.

Due to the constraints of manpower, time, and funding, this study took convenient sampling and only collected cases in the Yunlin county of Taiwan, and Yunlin county belongs to a rural city, so this study can only represent the results of these cases; it cannot be inferred to other metropolitan areas. It is suggested that future research can expand the scope of received cases and conduct a comparative analysis of rural and urban areas, which will increase the breadth and depth of inferences. In addition, although the researcher entered the study field as an intern, and the interviewees were willing to cooperate with the interviews, after all, the researcher was an outsider, and did not have familiar relationships with residents. Residents may have some reservations in answering questions, which is also one of the limitations of this research. This study is a cross-sectional study and cannot see the dynamic changes in place identity and life adaptation. It is suggested that in the future, a longitudinal cohort study can be conducted to understand the change in place identity and the adaptation to life of the residents of the institution in the time axis.

## Figures and Tables

**Table 1:** Personal characteristics *n* = 120.

Variables		*N*	%
Gender	Male	58	48.3
	Female	62	51.7
Age	M ± SD	78.8 ± 9.1	
	65–74	47	39.2
	75–84	35	29.2
	≥85	38	31.6
Marital Status	Married	40	33.3
	Unmarried/widowed/divorced	80	66.7
Level of Education	No formal education	36	30.0
	Primary school	55	45.8
	Junior high school or above	29	24.2
Economic status	Well off	21	17.5
	generally	99	82.5
Religion	No	6	5.0
	Yes	114	95
Check-in institution time	M ± SD	31.8 ± 33.7	
	<1 year	36	30.0
	1–5 (exclude) years	66	55.0
	≥5 years	18	15.0
Experience of staying in other institutions	No	102	85.0
	Yes	18	15.0
ADLs score	M ± SD	62.9 ± 24.1	
	≥91	15	12.5
	61–90	46	38.4
	≤60	59	49.1
IADLs score	M ± SD	5.7 ± 4.9	
Number of chronic diseases	M ± SD	1.84 ± 1.0	
	0	4	3.3
	1	42	35.0
	2	56	46.7
	≥3	18	15.0

**Table 2:** Distribution of Place Identity Scale and Life Adaptation Scale Score.

Items	Range	Mean	SD
Overall place identity scale	15–75	47.7	9.3
Sense of belonging	5–25	17.7	3.1
Sense of caring	5–25	15.6	3.8
Sense of participation	5–25	14.3	4.3
Overall life adaptation scale	25–100	79.6	9.22
Environmental structure	6–24	18.1	2.44
Life and accessories	6–24	16.9	2.97
Care management	6–24	21.0	3.49
Interpersonal social relationship	7–28	23.7	3.31

**Table 3:** Analysis between personal characteristics of residents and the scales of place identity *n* = 120.

Item	*N*	Overall Scale		Sense of Belonging		Sense of Caring		Sense of Participation	
		M ± SD	t/F	M ± SD	t/F	M ± SD	t/F	M ± SD	t/F
**Gender**									
Male	58	48.4 ± 9.5	0.65	18.0 ± 3.0	1.11	16.0 ± 3.5	0.88	14.4 ± 4.5	0.03
Female	62	47.0 ± 9.2		17.3 ± 3.2		15.3 ± 4.0		14.3 ± 4.3	
**Age**									
➀ 65–74	47	49.3 ± 10.2	1.35	18.0 ± 3.1	0.88	16.2 ± 4.0	1.05	15.0 ± 4.6	0.34
➁ 75–84	35	45.9 ± 7.3		17.1 ± 2.9		15.0 ± 3.0		13.7 ± 3.6	
➂ ≥85	38	47.3 ± 9.6		17.9 ± 3.3		15.5 ± 4.0		14.0 ± 4.6	
**Marital Status**									
Unmarried/widowed/divorced	80	47.3 ± 8.7	0.49	17.7 ± 3.1	0.13	15.4 ± 3.5	0.74	14.2 ± 4.1	0.23
Married	40	48.5 ± 10.5		17.9 ± 3.2		16.1 ± 4.2		14.0 ± 4.9	
**Education**									
No formal education	36	46.6 ± 9.6	0.75	17.6 ± 3.5	0.24	15.0 ± 4.1	1.14	14.1 ± 4.5	0.09
Primary school	55	47.4 ± 8.5		17.7 ± 2.8		15.6 ± 3.5		14.4 ± 3.9	
Junior high school or above	29	49.4 ± 10.4		18.1 ± 3.2		16.4 ± 3.8		14.6 ± 5.1	
**Economic status**									
Well off	21	46.7 ± 7.3	3.64	17.2 ± 3.4	0.75	16.0 ± 2.8	0.18	13.5 ± 3.3	0.86
Generally	99	47.9 ± 9.7		17.8 ± 3.0		15.6 ± 3.9		14.5 ± 4.5	
**Religion**									
Yes	114	48.7 ± 9.3	0.84	17.7 ± 3.1	0.80	15.6 ± 3.7	0.00	14.4 ± 4.3	0.14
No	6	48.2 ± 13.8		18.8 ± 4.0		15.7 ± 4.9		13.7 ± 5.9	
**Check-in institution time**									
➀ <1 year	36	44.5 ± 7.0	3.79 * ➂ > ➀	17.6 ± 2.5	0.65	14.3 ± 2.8	5.32 ** ➂ > ➀	12.7 ± 3.2	4.11 * ➂ > ➀
➁ 1–5 (exclude) years	66	48.3 ± 10.5		17.6 ± 3.7		15.8 ± 4.2		14.9 ± 4.6	
➂ ≥5 years	18	51.7 ± 6.6		18.5 ± 1.2		17.6 ± 2.7		15.6 ± 4.4	
**Experience of other** **institutions**									
Yes	18	46.6 ± 7.5	1.99	18.1 ± 3.0	0.22	15.5 ± 3.9	0.03	12.9 ± 3.7	2.20
No	102	47.9 ± 9.6		17.7 ± 3.1		15.7 ± 3.7		14.6 ± 4.4	
**ADLs**									
➀ 1 ≤ 60	59	44.3 ± 7.1	8.67 *** ➁ > ➀	17.2 ± 2.7	1.56	14.4 ± 3.0	7.5 ** ➁ > ➀	12.7 ± 3.5	9.09 *** ➁ > ➀ ➂ > ➀
➁ 61–90	46	51.1 ± 9.4		18.3 ± 3.2		16.9 ± 3.9		15.9 ± 4.4	
➂ ≥91 points	15	50.5 ± 12.2		17.9 ± 4.1		16.7 ± 4.3		15.9 ± 4.9	
**Number of chronic diseases**									
➀ 0	4	57.0 ± 13.8	4.33 **	19.8 ± 4.5	0.96	17.5 ± 5.5	3.79 * ➁ > ➂	19.8 ± 5.6	4.81 ** ➀ > ➂ ➀ > ➃
➁ 1	42	50.6 ± 10.8		18.0 ± 3.6		17.0 ± 4.2		15.5 ± 4.8	
➂ 2	56	45.5 ± 7.5		17.4 ± 2.8		14.7 ± 3.1		13.4 ± 3.6	
➃ ≥ 3	18	45.7 ± 7.1		17.6 ± 2.3		14.8 ± 3.0		13.2 ± 3.8	

P.S. * *p* < 0.05, ** *p* < 0.01, *** *p* < 0.001.

**Table 4:** Analysis between personal characteristics of residents and the scales of life adaptation *n* = 120.

Item	*n*	Overall Scale		Environmental Structure		Life and Accessories		Care Management		Interpersonal Social Relationship	
		M ± SD	t/F	M ± SD	t/F	M ± SD	t/F	M ± SD	t/F	M ± SD	t/F
**Gender**											
Male	58	80.2 ± 8.6	0.45	18.2 ± 2.7	0.41	17.1 ± 2.9	0.72	21.6 ± 3.2	0.50	23.6 ± 3.0	0.01
Female	62	79.1 ± 9.8		17.9 ± 2.2		16.7 ± 3.1		20.8 ± 3.8		23.7 ± 3.6	
**Age**											
➀ 65–74	47	81.2 ± 10.0	1.47	18.3 ± 2.9	0.61	17.7 ± 3.1	4.22 * ➀ > ➁	21.0 ± 3.2	0.00	24.1 ± 3.1	0.95
➁ 75–84	35	77.7 ± 8.2		17.7 ± 2.0		15.9 ± 2.1		21.0 ± 3.4		23.1 ± 3.4	
➂ ≥85	38	79.5 ± 9.0		18.1 ± 2.2		16.8 ± 3.2		21.0 ± 4.0		23.7 ± 3.5	
**Marital Status**											
Unmarried/widowed/ divorced	80	79.8 ± 8.8	0.05	18.0 ± 2.3	0.28	16.7 ± 2.9	1.34	21.4 ± 3.2	2.27	23.8 ± 3.3	0.22
Married	40	79.4 ± 10.2		18.2 ± 2.7		17.3 ± 3.2		20.4 ± 4.0		23.5 ± 3.4	
**Education**											
No formal education	36	79.8 ± 10.3	0.10	18.0 ± 2.5	0.75	16.7 ± 3.1	0.28	21.7 ± 3.9	1.04	23.5 ± 3.6	0.14
Primary school	55	79.9 ± 8.3		18.3 ± 2.4		16.8 ± 2.6		20.9 ± 3.2		23.8 ± 3.1	
Junior high school or above	29	79.0 ± 9.8		17.7 ± 2.4		17.2 ± 3.5		20.5 ± 3.5		23.6 ± 3.4	
**Economic status**											
Well off	21	79.3 ± 6.5	0.03	17.4 ± 1.6	1.71	16.4 ± 2.5	0.73	21.6 ± 3.3	0.62	24.0 ± 2.4	0.18
Generally	99	79.7 ± 9.7		18.2 ± 2.6		17.0 ± 3.1		20.9 ± 3.5		23.6 ± 3.5	
**Religion**											
Yes	114	79.6 ± 9.3	0.11	18.0 ± 2.4	0.64	16.9 ± 3.0	0.11	21.0 ± 3.5	0.21	23.7 ± 3.3	0.01
No	6	80.8 ± 8.8		18.8 ± 2.6		16.5 ± 2.6		21.7 ± 2.9		23.8 ± 3.1	
**Check-in institution time**											
➀ <1 year	36	77.5 ± 7.5	2.97	17.6 ± 2.5	1.83	17.1 ± 3.1	2.56	20.2 ± 3.1	1.76	23.9 ± 3.4	0.24
➁ 1–5 (exclude) years	66	81.5 ± 10.3		18.4 ± 2.7		17.2 ± 3.1		21.5 ± 3.3		23.7 ± 3.4	
➂ ≥5 years	18	77.3 ± 7.0		17.5 ± 0.6		15.4 ± 1.8		20.8 ± 4.7		23.2 ± 3.1	
**Experience of other** **institutions**											
Yes	18	79.4 ± 9.5	0.50	18.6 ± 2.5	0.88	17.2 ± 2.6	0.28	22.1 ± 3.1	1.30	23.2 ± 2.8	0.39
No	102	81.1 ± 7.4		18.0 ± 2.4		16.8 ± 3.0		20.8 ± 3.5		23.8 ± 3.4	
**ADLs**											
➀ 1≤60	59	78.3 ± 8.2	1.30	17.4 ± 1.7	6.15 ** ➂ > ➀	16.2 ± 2.6	3.13	21.2 ± 3.2	0.43	23.4 ± 3.3	0.64
➁ 61–90	46	81.1 ± 9.1		18.3 ± 2.8		17.6 ± 2.9		21.0 ± 3.2		24.1 ± 3.2	
➂ ≥91 points	15	80.6 ± 12.8		19.7 ± 3.0		17.2 ± 4.1		20.3 ± 3.6		24.4 ± 4.0	
**Number of chronic** **diseases**											
➀ 0	4	81.3 ± 10.2	5.35 ** ➁ > ➂	19.5 ± 3.0	8.23 *** ➁ > ➂ ➁ > ➃	19.8 ± 4.4	5.49 ** ➁ > ➂ ➁ > ➃	19.3 ± 1.9	5.35 ** ➁ > ➂	22.8 ± 3.5	1.23
➁ 1	42	83.9 ± 9.4		19.3 ± 3.0		18.0 ± 2.9		22.1 ± 2.8		24.5 ± 3.2	
➂ 2	56	77.0 ± 8.3		17.3 ± 1.9		16.3 ± 2.8		20.2 ± 3.6		23.3 ± 3.5	
➃≥3	18	77.6 ± 8.2		17.2 ± 0.5		15.6 ± 2.2		21.4 ± 4.1		23.4 ± 3.0	

P.S. * *p* < 0.05, ** *p* < 0.01, *** *p* < 0.001.

**Table 5:** The relationship between the place identity and the life adaptation *n* = 120.

Item	Environmental Structure		Life and Accessories		Care Management		Interpersonal Social Relationship		Overall of Life Adaptation	
	r	*p*	r	*p*	r	*p*	r	*p*	r	*p*
Sense of belonging	0.28	0.002 **	0.36	0.000 ***	0.17	0.06	0.12	0.18	30	0.001 **
Sense of caring	0.27	0.003 **	0.34	0.000 ***	0.05	0.56	0.16	0.08	0.26	0.004 **
Sense of participation	0.33	0.000 ***	0.29	0.001 **	−0.15	0.11	0.002	0.98	0.13	0.17
Overall of place identity	0.36	0.000 ***	0.39	0.000 ***	0.01	0.90	0.11	0.24	0.26	0.004 **

Notes: ** *p* < 0.01; *** *p* < 0.01.

## Data Availability

The data presented in this study are available on request from the corresponding author. The data are not publicly available to retain participant privacy.

## References

[1] United Nations World Population Prospects: The 2017 Revision. https://esa.un.org/unpd/wpp/Download/Probabilistic/Population/.

[2] Department of Household Registration, Ministry of the Interior Taiwan Demographics Data in December 2020. https://www.ris.gov.tw/app/portal/346.

[3] National Development Council Handbook of Important Statistics in December 2018. https://www.ndc.gov.tw/Content_List.aspx?n=507E4787819DDCE6.

[4] Diwan S., Lee S.E., Sen S. (2011). Expectations of filial obligation and their impact on preferences for future living arrangements of middle-aged and older Asian Indian immigrants. J. Cross-Cult. Gerontol..

[5] Chen Y.-J., Wang S.-C. (2016). Chinese concept of filial piety and decision-making of intergenerational living arrangements for the elderly. Humanit. Soc. Sci. Newsl. Q..

[6] Yasuda T., Iwai N., Chin-Chun Y., Guihua X. (2011). Intergenerational coresidence in China, Japan, South Korea and Taiwan: Comparative analyses based on the East Asian Social Survey 2006. J. Comp. Fam. Stud..

[7] Liu J., Chiag L. (2019). The trend of living conditions and institutional care needs of the elderly in Taiwan. Public Gov. Q..

[8] Ministry of Health and Welfare Aged and Long-Term Care Statistics. https://dep.mohw.gov.tw/DOS/cp-4226-45154-113.html.

[9] Ministry of the Interior, Republic of China (Taiwan) Analysis on the Statistics of Household Registration in 2021. https://www.moi.gov.tw/News_Content.aspx?n=9&sms=9009&s=212975.

[10] Ministry of Health and Welfare Overview of Long-Term Care and Nursing Institutions for the Elderly. https://dep.mohw.gov.tw/dos/cp-2977-13854-113.html.

[11] Zamanzadeh V., Rahmani A., Pakpour V., Chenoweth L.L., Mohammadi E. (2017). Psychosocial changes following transition to an aged care home: Qualitative findings from Iran. Int. J. Older People Nurs..

[12] Chang S.J. (2013). Lived experiences of nursing home residents in Korea. Asian Nurs. Res..

[13] Lin L.J. (2013). The factors affecting the relocation adjustment for the residents: A case study of a long term care facility in Taipei. Leis. Soc. Res..

[14] Lin Y.P., Chang H.Y. (2017). Application of transitional theory to explore the related factors of life adaptation in elderly who relocated to long-term care institutions. Macau J. Nurs..

[15] Bekhet A.K., Zauszniewski J.A. (2014). Individual characteristics and relocation factors affecting adjustment among relocated American and Egyptian older adults. Issues Ment. Health Nurs..

[16] Sun C., Ding Y., Cui Y., Zhu S., Li X., Chen S., Zhou R., Yu Y. (2021). The adaptation of older adults’ transition to residential care facilities and cultural factors: A meta-synthesis. BMC Geriatics..

[17] Lewicka M. (2011). Place attachment: How far have we come in the last 40 years?. J. Environ. Psychol..

[18] Relph E.C. (1976). Place and Placelessness.

[19] Entrikin J.N. (1994). Place and region. Prog. Hum. Geogr..

[20] Tsaur S.-H., Sun C.-Y. (2009). Antecedents and consequences of place attachment. J. Geogr. Sci..

[21] Wei M.-H., Tsai Y.-H. (2016). The comparative study of parents’ evaluation of school and family adjustment between disabled children and typical developing children. Yu Da Acad. J..

[22] Lee H.-M., Su S.H., Jeng B.-J. (2019). The effects of positive happiness intervention program on children’s life adaptation. Bull. Chung Hwa Univ. Med..

[23] Chen Y.-H. (2017). A Case Study of Conflict Experience and Adaptation among the Residents in a Senior Congregate Housing in Kaohsiung. Master’s Thesis.

[24] Chen T.Z., Lee P.L., Huang C.K., Kung Y.L. (2015). The relationship between life adaptation, life satisfaction and loneliness of elderly. J. Crisis. Manag..

[25] Hagerty B.M., Williams R.A., Coyne J.C., Early M.R. (1996). Sense of belonging and indicators of social and psychological functioning. Arch. Psychiatr. Nurs..

[26] Wu C.-C., Huang H.-M., Gao C.-C., Gau B.-H. (2010). Autonomy and its related factors among older people in long-term care facilities. J. Long-Term Care.

[27] Malpas J. (2009). Place and Experience: A Philosophical Topography.

[28] McShane S.L., von Glinow M.A. (2000). Organizational Behavior.

[29] James I., Blomberg K., Kihlgren A. (2014). A meaningful daily life in nursing homes-a place of shelter and a space of freedom: A participatory appreciative action reflection study. BMC Nurs..

[30] Tseng H.-Y., Leu Y.-R., Chiang J.-Y. (2019). Exploring family caregiving images: Daily life practice of elderly married couples relocating to a residential care facility. Taiwan Geriatr. Gerontol..

[31] Pretty G.H., Chipuer H.M., Bramston P. (2003). Sense of place amongst adolescents and adults in two rural Australian towns: The discriminating features of place attachment, sense of community and place dependence in relation to place identity. J. Environ. Psychol..

[32] Fleming A., Kydd A., Stewart S. (2017). Care homes: The developing ideology of a homelike place to live. Maturitas.

[33] Chen H.-M., Huang M.-C. (2013). The promotion of autonomy for disabled elderly toward involuntary institutional stay. J. Long-Term Care.

[34] Lin T.L., Mao H.-F., Wang S.-C., Hu M.-H. (2017). Psychosocial adaptation of the elderly in the nursing home. J. Long-Term Care.

[35] Tsai H.Y., Chang Y.L., Chan H.S. (2015). A change for vision-to review the quality of elder day care service in the sight of service users. J. Community Work Community Stud..

[36] Tasi Y.-Y., Hsu Y.-C., Yang T. (2013). An investigation of the relationships between physical function, social activity and loneliness of the elderly living in the nursing homes. J. Long-Term Care.

